# Identification of miR-30e* Regulation of Bmi1 Expression Mediated by Tumor-Associated Macrophages in Gastrointestinal Cancer

**DOI:** 10.1371/journal.pone.0081839

**Published:** 2013-11-28

**Authors:** Hidetaka Sugihara, Takatsugu Ishimoto, Masayuki Watanabe, Hiroshi Sawayama, Masaaki Iwatsuki, Yoshifumi Baba, Yoshihiro Komohara, Motohiro Takeya, Hideo Baba

**Affiliations:** 1 Department of Gastroenterological Surgery, Graduate School of Medical Science, Kumamoto University, Kumamoto, Japan; 2 Department of Cell Pathology, Graduate School of Medical Science, Kumamoto University, Kumamoto, Japan; Sun Yat-sen University Medical School, China

## Abstract

Bmi1 is overexpressed in a variety of human cancers including gastrointestinal cancer. The high expression level of Bmi1 protein is associated with poor prognosis of gastrointestinal cancer patients. On the other hand, tumor-associated macrophages (TAMs) contribute to tumor growth, invasion, and metastasis by producing various mediators in the tumor microenvironment. The aim of this study was to investigate TAM-mediated regulation of Bmi1 expression in gastrointestinal cancer. The relationship between TAMs and Bmi1 expression was analyzed by immunohistochemistry and quantitative real-time PCR (qRT-PCR), and results showed a positive correlation with tumor-infiltrating macrophages (CD68 and CD163) and Bmi1 expression in cancer cells. Co-culture with TAMs triggered Bmi1 expression in cancer cell lines and enhanced sphere formation ability. miRNA microarray analysis of a gastric cancer cell line co-cultured with macrophages was conducted, and using *in silico* methods to analyze the results, we identified miR-30e* as a potential regulator of Bmi1 expression. Luciferase assays using miR-30e* mimic revealed that Bmi1 was a direct target for miR-30e* by interactions with the putative miR-30e* binding sites in the Bmi1 3′ untranslated region. qRT-PCR analysis of resected cancer specimens showed that miR-30e* expression was downregulated in tumor regions compared with non-tumor regions, and Bmi1 expression was inversely correlated with miR-30e* expression in gastric cancer tissues, but not in colon cancer tissues. Our findings suggest that TAMs may cause increased Bmi1 expression through miR-30e* suppression, leading to tumor progression. The suppression of Bmi1 expression mediated by TAMs may thus represent a possible strategy as the treatment of gastrointestinal cancer.

## Introduction

Bmi1 is a member of the polycomb-repressive complex 1 with an essential role in maintaining chromatin silencing [1,2]. Bmi1 plays a function in the self-renewal of neuronal and hematopoietic stem cells through repression of the INK4a/ARF locus [3-6]. Additionally, Bmi1 is expressed in intestinal stem cells and implicated in maintaining the small intestine epithelium [7]. Bmi1 was first identified as an oncogene that cooperates with c-myc during mouse lymphomagenesis, and is overexpressed in a variety of human cancers, including gastrointestinal cancer [8-10]. Furthermore, the expression level of Bmi1 protein is associated with poor prognosis of gastrointestinal cancer patients [9,10]. However, the mechanism underlying Bmi1 regulation in cancer cells is largely unknown.

Solid tumors consist of cancer cells and various types of stromal cells, fibroblasts, endothelial cells and hematopoietic cells, mainly macrophages and lymphocytes. Macrophages have functional plasticity and are described by two distinct polarization states: classically-activated (M1) and alternatively-activated (M2) macrophage phenotypes. Previous studies revealed that M1- and M2-polarized macrophages play different functional roles in the tumor microenvironment [11,12]. M1-polarized macrophages have generally antigen presenting functions and tumoricidal activity. In contrast, M2-polarized macrophages play a role in the response to parasites, wound healing, tissue remodeling, and promote the growth and vascularization of tumors. In many human cancers, tumor-associated macrophages (TAMs) contribute to tumor growth, invasion, and metastasis by secreting various mediators, so it was proposed that TAMs were predominantly polarized to M2 macrophage phenotype [13-17]. On the other hand, more recent studies demonstrated that macrophages were very plastic cells, and their epigenetic changes reprogramed TAMs from an M2 to an M1-like phenotype in tumors [17,18].

MicroRNAs (miRNAs) are non-coding RNAs (21–23 nucleotides) that bind imperfectly to the 3′ untranslated region (UTR) of their target mRNAs to repress their translation. miRNAs have been found to target various oncogenes and tumor suppressors, and emerging evidence suggests that dysregulation of miRNAs is involved in the pathogenesis of many cancers [19,20].

To explore the regulation of Bmi1 expression in cancer cells, we examined a possible correlation between Bmi1 expression in gastrointestinal cancer cells and infiltrating macrophages in the tumor microenvironment, and investigated the mechanism underlying the regulation of Bmi1 expression. Here we demonstrate that miR-30e* mediated by TAMs directly regulates Bmi1 expression in gastrointestinal cancer.

## Materials and Methods

### Cell culture and treatment

The cell lines AGS, NUGC4, COLO201, and THP-1 were cultured in 5% CO_2_ at 37°C in RPMI 1640 supplemented with 10% fetal bovine serum (FBS). HCT116 cells were cultured under 5% CO_2_ at 37°C in Dulbecco’s modified Eagle’s medium-nutrient mixture F-12 (Sigma, St. Louis, MO, USA) supplemented with 10% FBS. The cell lines were obtained from the Japanese Collection of Research Bioresources Cell Bank and Riken BioResource Center Cell Bank.

### Immunohistochemistry (IHC) and scoring

Sample processing and IHC procedures were performed as previously described[21]. Endogenous peroxidase activity was blocked using 3% hydrogen peroxide. The sections were incubated first with diluted antibodies, followed by incubation with biotin-free horseradish peroxidase-labeled polymer from the Envision Plus detection system (Dako, Glostrup, Denmark). Positive reactions were visualized using diaminobenzidine solution, and counterstained with Meyer’s hematoxylin. As negative control, mouse primary antibodies were used and no positive stains were seen. All IHC staining was scored independently by two pathologists. Nuclear Bmi1 and cytoplasmic CD68 and CD163 expressions were interpreted according to the guidelines published in the previous study. For nuclear Bmi1 and cytoplasmic CD68 and CD163, we scored the positive staining results in categories from 0 to 3+ as follows: 0, no staining; 1+, 1–25% of the specimen stained; 2+, 26–50%; and 3+, >50%. A score of 3+ was considered to be a positive IHC result.

### Antibodies for IHC and immunoblotting analyses

The following antibodies were used for IHC analysis: a mouse monoclonal antibody specific for human Bmi1 (1:100 dilution; Abcam, Cambridge, UK), a mouse monoclonal antibody specific for human CD68 (1:100 dilution; Dako, Glostrup, Denmark), and a mouse monoclonal antibody specific for human CD163 (1:100 dilution; Novocastra, Newcastle, UK). The following antibodies were used for immunoblot analysis: a mouse monoclonal antibodies to Bmi1 (1:1000), and a rabbit polyclonal antibody for human β-actin (1:1000; Cell Signaling Technology).

### RNA and miRNA isolation

Total RNA, including miRNA, was isolated from cell lines using a mirVana miRNA Isolation Kit (Ambion, Austin, TX, USA), and eluted into 100 μl of heated elution solution, according to the manufacturer’s protocol. miRNAs were extracted from formalin-fixed paraffin-embedded gastrointestinal cancer tissues and their matched adjacent normal gastrointestinal epithelia using a RecoverAll Total Nucleic Acid Isolation Kit for FFPE (Ambion), according to the manufacturer’s instructions. The purity and concentration of all RNA samples were evaluated by their absorbance ratio at 260/280 nm, determined using a NanoDrop ND-1000 spectrophotometer (NanoDrop Technologies, Rockland, DE, USA).

### THP-1 macrophage preparation and co-culture assay

THP-1 cells were seeded in the transwell inserts (3540, Corning) for 6-well plates (1 × 10^6^ cells/well). For preparation of M1-polarized THP-1 macrophages, 320 nM phorbol myristate acetate (PMA) was added to THP-1 cells for 6 h, followed by PMA plus 20 ng/ml interferon (IFN)-γ and 100 ng/ml lipopolysaccharide for the following 18 h. For preparation of M2-polarized THP-1 macrophages, 320 nM PMA was added to THP-1 cells for 6 h, followed by PMA plus 20 ng/ml interleukin (IL)-4/IL-13 for the following 18 h. After three washes to remove cytokines, M1- or M2-polarized THP-1 macrophages were co-cultured in upper inserts with AGS or HCT116 cells in 6-well plates (1 × 10^5^ cells/well) without direct contact, in each medium without 10% FBS as described above. After 24 h of co-culture, the upper inserts containing macrophages were discarded. AGS and HCT116 cells were washed and used for subsequent experiments.

### Sphere culture

As described above, M1- or M2-polarized THP-1 macrophages was prepared. After three washes to remove cytokines, M1- or M2-polarized THP-1 macrophages were co-cultured in upper inserts with AGS and HCT116 cells (1 × 10^4^ cells/well) non-adhesively in 6-well plates (3471, Corning) without direct contact, coated with thin agarose at a density of 2 × 10^4^/mm^3^ in serum-free DMEM/F12 medium (Invitrogen) containing 1% N2 (Gibco), 2% B27 (Gibco), 20 ng/ml human fibroblast growth factor (FGF)-2 (Sigma, St. Louis, MO), and 20 ng/ml epidermal growth factor (EGF) (Sigma). Each treatment was carried out in triplicate. The culture medium was changed every other day until sphere formation. After 10 days, the spheres were collected.

### Macrophage culture and co-culture assay

Peripheral blood mononuclear cells were obtained from healthy volunteer donors. CD14+ monocytes were isolated using CD14 microbeads (Miltenyi Biotec, Bergisch Gladbach, Germany). Monocytes were plated in 6-well plates (1 × 10^5^/well) and cultured with granulocyte M-CSF (2 ng/mL) (Wako, Tokyo, Japan) for five days to induce immature macrophages. After washes with PBS, cells were stimulated with IFN-γ (1 ng/mL) (PeproTech, Rocky Hill, NJ, USA) to induce M1 macrophages. Monocytes were plated and cultured with M-CSF (100 ng/mL) (Wako) for five days to induce immature macrophages. After washes with PBS, cells were stimulated with IL-10 (10 ng/mL) (PeproTech) to induce M2 macrophages. Media from M1- or M2-polarized macrophage cultures was collected and transferred into 6-well plates containing AGS and HCT116 cells (1×10^4^ cells/well). After 24 h of co-culture, AGS and HCT116 cells were washed and used for subsequent experiments. 

### miRNA microarray

Cyanine-3 (Cy3) labeled cRNA was prepared from 100 ng RNA using Agilent's miRNA Complete Labeling and Hyb Kit (p/n 5190-0456) according to the manufacturer's instructions. Agilent Human 8 x 60K miRNA Array was performed on the two pooled samples. Hybridization was carried out according to the instructions of the Agilent's miRNA Complete Labeling and Hyb Kit. Slides were scanned immediately after washing on the Agilent DNA Microarray Scanner (G2505C) using one color scan setting for 8x60k array slides (Scan Area 61x21.6 mm, Scan resolution 5um, Dye channel is set to Green and Green PMT is set to 100%). The scanned images were analyzed with Feature Extraction Software 10.7.3.1 (Agilent) using default parameters (protocol miRNA_107_Sep09). Probe intensities were normalized using GeneSpring 12.0 through percentile shift normalization. Differentially expressed miRNAs were identified through Fold Change filtering. Microarray data have been deposited in GEO (accession no. GSE50601; http://www.ncbi.nlm.nih.gov/geo/query/acc.cgi?acc=GSE50601).

### Quantitative real-time reverse transcription-polymerase chain reaction (qRT-PCR)

The expression levels of miR-30e* were determined by TaqMan qRT-PCR using TaqMan miRNA assay kits (Ambion), according to the manufacturer’s protocol, as described previously. miR-30e* expression was normalized to the expression of RNU6B small nuclear RNA. Expression levels of Bmi1 were quantified by Probes Master qRT-PCR using a LightCycler 480 Probes Master (Roche Diagnostics, Mannheim, Germany) and normalized to glyceraldehyde-3-phosphate dehydrogenase. All qRT-PCR reactions were run using the LightCycler 480 System II (Roche Diagnostics). The relative amounts of miR-30e* and Bmi1 were measured with the 2^-ΔΔCT^ method. All qRT-PCR reactions were performed in triplicate.

### Transfection of miRNA

Cells were transfected with 5 nM mimic or inhibitor miR-30e* (Applied Biosystems, Foster City, CA, USA) using Lipofectamine RNAiMax transfection reagent (Invitrogen, Carlsbad, CA, USA), according to the manufacturer’s instructions. The specificity of the transfection was verified using a negative control mimic (Applied Biosystems). The expression levels of miR-30e* were quantified 48 h after transfection, and the cells were used for subsequent experiments.

### Generation of Bmi1 3'UTR mutants

Vectors containing mutated miR-30e* target sequences in the human Bmi1 3'UTR were introduced by site-directed mutagenesis using the following PCR primers: 5′- ccUAUGGACGU-UAAUUGAAAa -3′ for Luc-Bmi1-wild-type, and 5′- ccUAUGGACGU-UAUGACUUUa -3′ for Luc-Bmi1-mutant.

### Luciferase assay

AGS cells were plated in 96-well plates and transfected with MultiFectam (Promega) using the pMIR-REPORT™ Luciferase miRNA Expression Reporter Vector containing firefly luciferase under the control of a mammalian promoter/terminator system. A miRNA target cloning region was included downstream of the luciferase translation sequence or empty vector (Invitrogen), and mimic control or mimic miR-30e* (Invitrogen). Reporter assays were performed 48 h after transfection with the Luc-Screen® System (Applied Biosystems) according to the manufacturer’s instructions. All experiments were conducted in triplicate.

### Western blot analysis

Cultured cells collected from 6-well plates were washed once in PBS and lysed in radioimmunoprecipitation buffer supplemented with protease/phosphatase inhibitor cocktail (Thermo Scientific, Tokyo, Japan). Protein samples were subjected to sodium dodecyl sulfate-polyacrylamide gel electrophoresis and transferred to a nitrocellulose membrane, and the membrane was incubated with primary antibodies. Signals were detected by incubation with secondary antibodies using the ECL Detection System (GE Healthcare, Little Chalfont, UK).

### Patients and tissue samples

Primary gastrointestinal carcinoma tissues and their matched adjacent normal gastrointestinal epithelia were obtained from 83 gastric cancer patients and 49 colon cancer patients who underwent gastrointestinal cancer resection without preoperative treatment at the Department of Gastroenterological Surgery, Kumamoto University Hospital from 2005 to 2008. Signed informed consent to participate was obtained from all patients. The study was approved by the medical ethics committee of Kumamoto University.

### Statistical analysis

All experiments were performed in triplicate and the data shown are representative of consistently observed results. Data are presented as the mean±standard deviation (SD). Chi-squared tests were used to assess the differences in proportion between Bmi1 expression and CD68/CD163 expression. Independent Student’s *t*-tests were used to compare continuous variables between the two groups, and Tukey-HSD procedure was used to compare continuous variables between the three groups. For the statistical analyses, we used the JMP (Version 9, SAS Institute) and the SAS software programs (Version 9.1, SAS Institute). A P value of < 0.05 was considered statistically significant.

## Results

### The expression of Bmi1 correlates with the levels of TAMs in gastrointestinal cancer tissues

**Figure 1:**
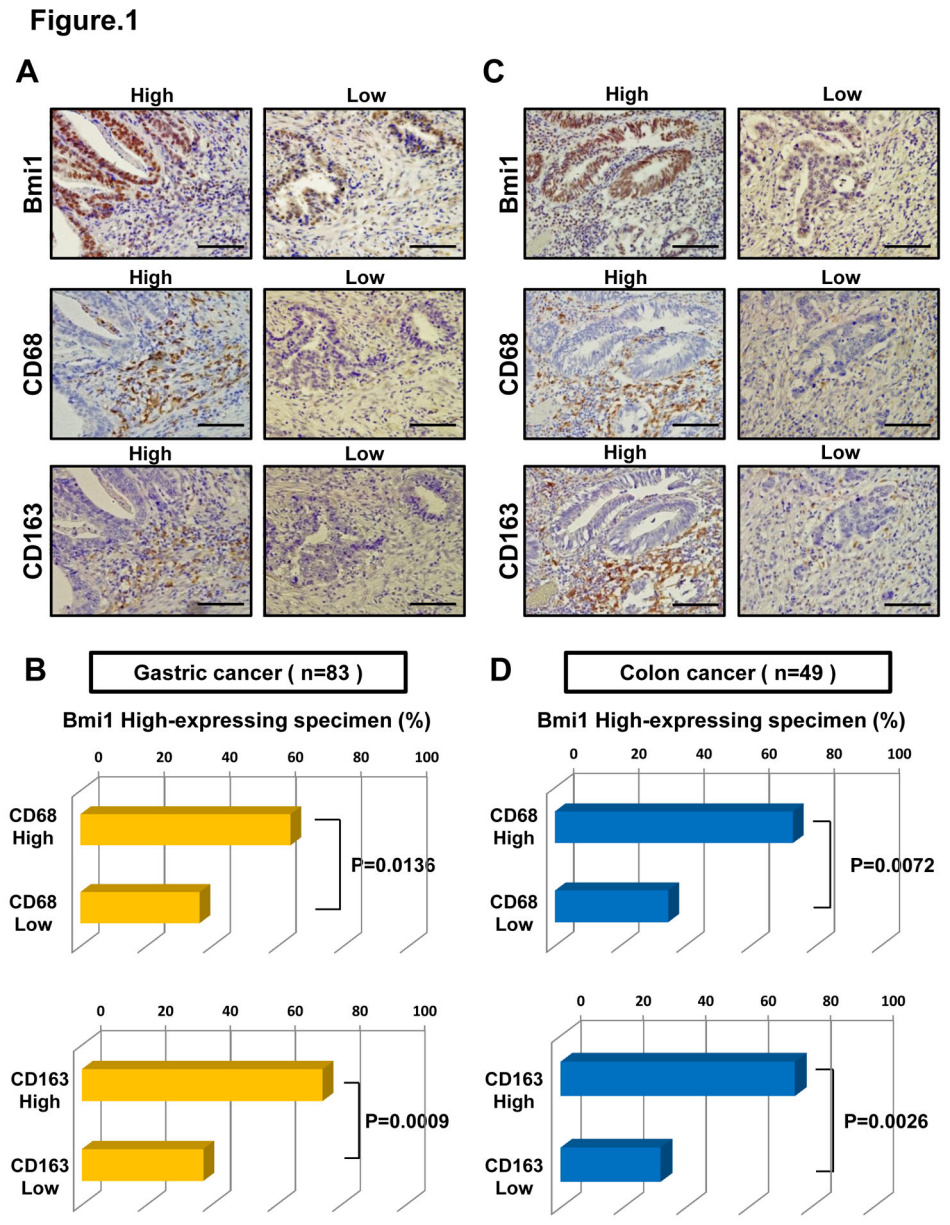
Relationship between the expression of Bmi1 and levels of TAMs. (**A**) Immunohistochemistry of Bmi1, CD68, and CD163 expression in 83 gastric cancer tissues. Scale bars, 100um. (**B**) The percentage of CD68/163 positive specimens in high Bmi1 expressing gastric cancer. There was a significant correlation between Bmi1 expression and CD68/163 expression (*P < 0.05, ***P < 0.001, respectively). (**C**) Immunohistochemistry of Bmi1, CD68, and CD163 expression in 49 colon cancer tissues. Scale bars, 100um. (**D**) The percentage of CD68/163 positive specimens in high Bmi1 expressing colon cancer. There was a significant correlation between these two groups (**P < 0.01, **P < 0.01, respectively).

TAMs contribute to tumor growth, invasion, and metastasis in many cancers by producing various mediators[13-17]. To determine whether the expression of Bmi1 in cancer cells correlates with the levels of TAMs, we examined Bmi1, CD68, and CD163 expression in gastrointestinal cancer tissues using IHC. CD68 staining was used to detect pan-macrophages, and the M2 population was evaluated using CD163, as described previously[22]. Results showed a positive relationship with Bmi1 and CD68/CD163 expression in gastric cancer (Figure 1A, C) and in colon cancer (Figure 1B, D). These results suggest that macrophages in tumor stroma may be involved in Bmi1 expression in gastrointestinal cancer cells.

Bmi1 expression is increased in gastrointestinal cancer cell lines co-cultured with M1- or M2-polarized THP-1 macrophages, leading to the acquired ability of sphere formation in 3D culture

**Figure 2:**
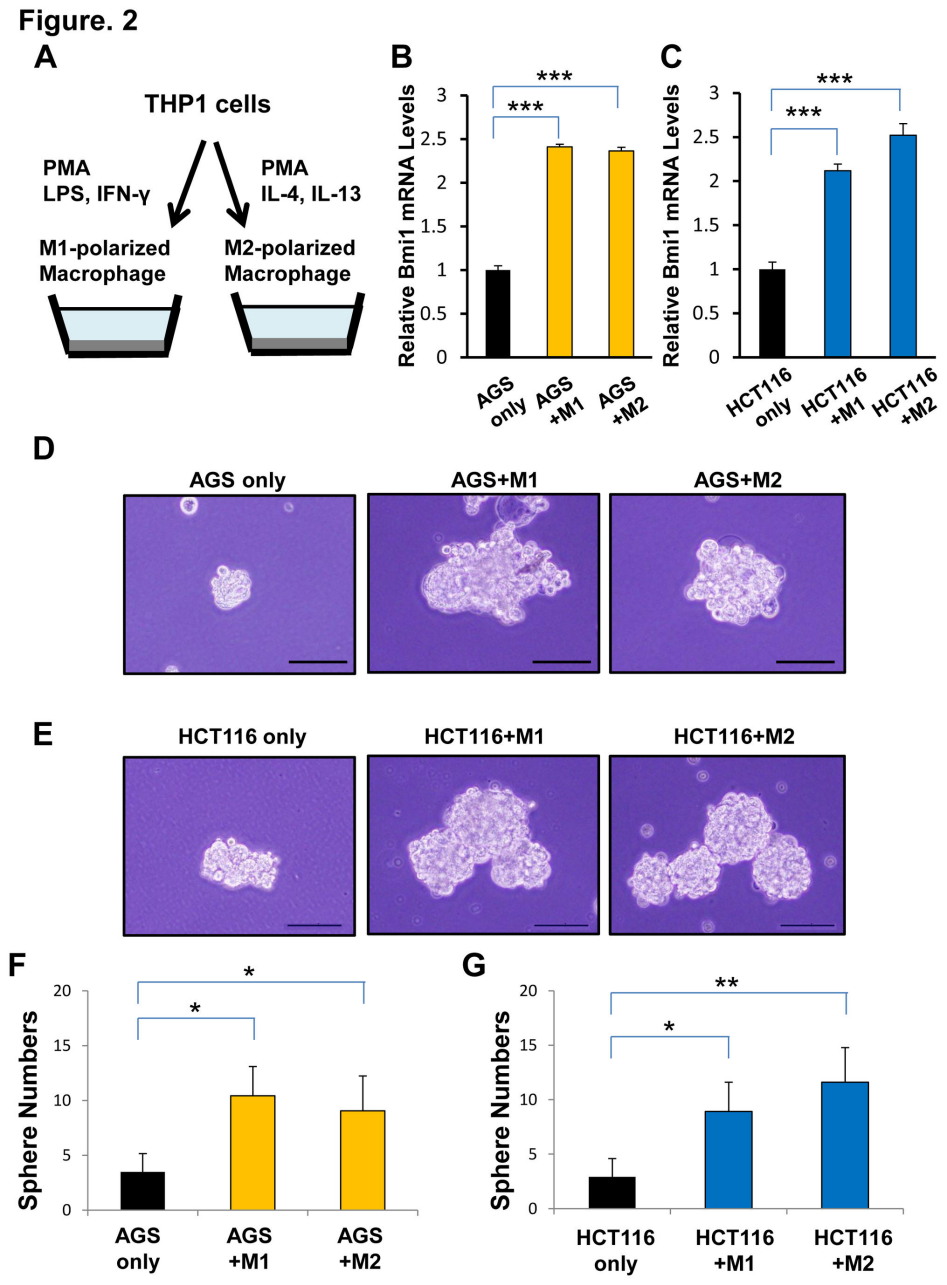
Bmi1 expression and sphere assay in gastrointestinal cancer cell lines co-cultured with M1- or M2-polarized THP-1 macrophages. (**A**) Cytokine production profile of M1- and-M2 polarized THP-1 macrophages. (**B**) qRT-PCR analysis of Bmi1 expression in AGS cells co-cultured with M1- and M2-polarized THP-1 macrophages, compared with Bmi1 expression in AGS cells only as a control group. Significantly higher Bmi1 expression was detected in co-cultured groups compared with the control group (***P < 0.001, ***P < 0.001, respectively). (**C**) qRT-PCR analysis of Bmi1 expression in HCT116 cells co-cultured with M1- and M2-polarized THP-1 macrophages, compared with Bmi1 expression in HCT116 cells only as a control group. Significantly higher Bmi1 expression was detected in co-cultured groups compared with the control group (***P < 0.001, ***P < 0.001, respectively). (**D**) Microscopic images of 3D sphere cultured AGS cells co-cultured with macrophages, compared with 3D sphere cultured AGS cells only as a control group. Scale bars, 100um. (**E**) Microscopic images of 3D sphere cultured HCT116 cells co-cultured with macrophages, compared with 3D sphere cultured HCT116 cells only as a control group. Scale bars, 100um. (**F**) Significantly higher sphere numbers were detected in co-cultured groups compared with the control group in AGS cells (*P < 0.05, *P < 0.05, respectively). (**G**) Significantly higher sphere numbers were detected in co-cultured groups compared with the control group in HCT116 cells (*P < 0.05, **P < 0.01, respectively).

We next performed an *in vitro* co-culture assay with M1- or M2-polarized THP-1 macrophages to examine if macrophages affect Bmi1 expression in cancer cells and cancer cell functions. As shown previously, THP-1 cells were differentiated into M1- or M2-polarized macrophages by distinct cytokines treatment (Figure 2A)[23]. qRT-PCR analysis revealed that Bmi1 expression was significantly increased in AGS and HCT116 cells co-cultured with both M1- and M2-polarized THP-1 macrophages (Figure 2B, C). Bmi1 is involved in the self-renewal capacity through repression of the INK4a-ARF locus, thus we hypothesized that gastrointestinal cells co-cultured with TAMs may possess the capacity for self-renewal through upregulating Bmi1 expression. To investigate the phenotype of gastrointestinal cells co-cultured with TAMs, we performed a 3D sphere culture grown in serum-free non-adherent culture in gastrointestinal cells co-cultured with M1- or M2-polarized THP-1 macrophages (Figure 2D, E). The sphere formation ability of gastrointestinal cells co-cultured with TAMs was enhanced (Figure 2F, G). These results suggested that TAMs upregulated Bmi1 expression and enhanced sphere formation.

### Identification of miRNAs regulating Bmi1 expression using cancer-related miRNA screening in gastric cancer cells

Several miRNAs are implicated in regulating the activities of cancer stem cells, including self-renewal and tumorigenicity[19,20]. We therefore tested the hypothesis that the regulation of Bmi1 expression in gastrointestinal cancer cells may be mediated by miRNAs using miRNA microarray analysis. We selected the top ten most downregulated miRNAs in gastrointestinal cells co-cultured with M1- or M2-polarized THP-1 macrophages compared with gastrointestinal cells alone (Table 1A, B). Using several online databases (miRanda, Diana, Targetscan, TargetMiner, miRbase), miR-30e-3p (miR-30e*) was the only candidate miRNA among all identified miRNAs found to directly target the Bmi1 3′ UTR. We therefore focused on miR-30e* for further analysis.

**Table 1:** Microarray analysis of 1360 miRNAs in AGS cell lines co-cultured with THP-1 macrophages.

**A**		**B**	
miRNA	fold change (M1 vs control)	miRNA	fold change (M2 vs control)
hsa-miR-3682	0.006816627	hsa-miR-3682	0.005809477
hsa-miR-30e-3p	0.011009754	hsa-miR-373-3p	0.015210649
hsa-miR-335	0.013768308	hsa-miR-192-3p	0.015870558
hsa-miR-335-3p	0.016194038	hsv1-miR-H6-3p	0.016751566
hsa-miR-373-3p	0.017847616	hsa-miR-1225-3p	0.017139628
hsa-miR-192-3p	0.01862193	hsa-miR-3676	0.017353492
hsa-miR-296-5p	0.019188985	hsa-miR-766	0.032502682
hsa-miR-1225-3p	0.020111009	hsa-miR-335-3p	0.324230407
hsa-miR-766	0.038137453	hsa-miR-769-5p	0.445738351
hsa-miR-769-5p	0.434152471	hsa-miR-30e-3p	0.54343376

(A) The top ten miRNAs downregulated in AGS cell lines co-cultured with M1-polarized macrophages compared with controls. (B) The top ten miRNAs downregulated in AGS cell lines co-cultured with M2-polarized macrophages compared with controls.

### miR-30e* suppresses Bmi1 expression in gastrointestinal cells and directly targets the Bmi1 3′ UTR

**Figure 3:**
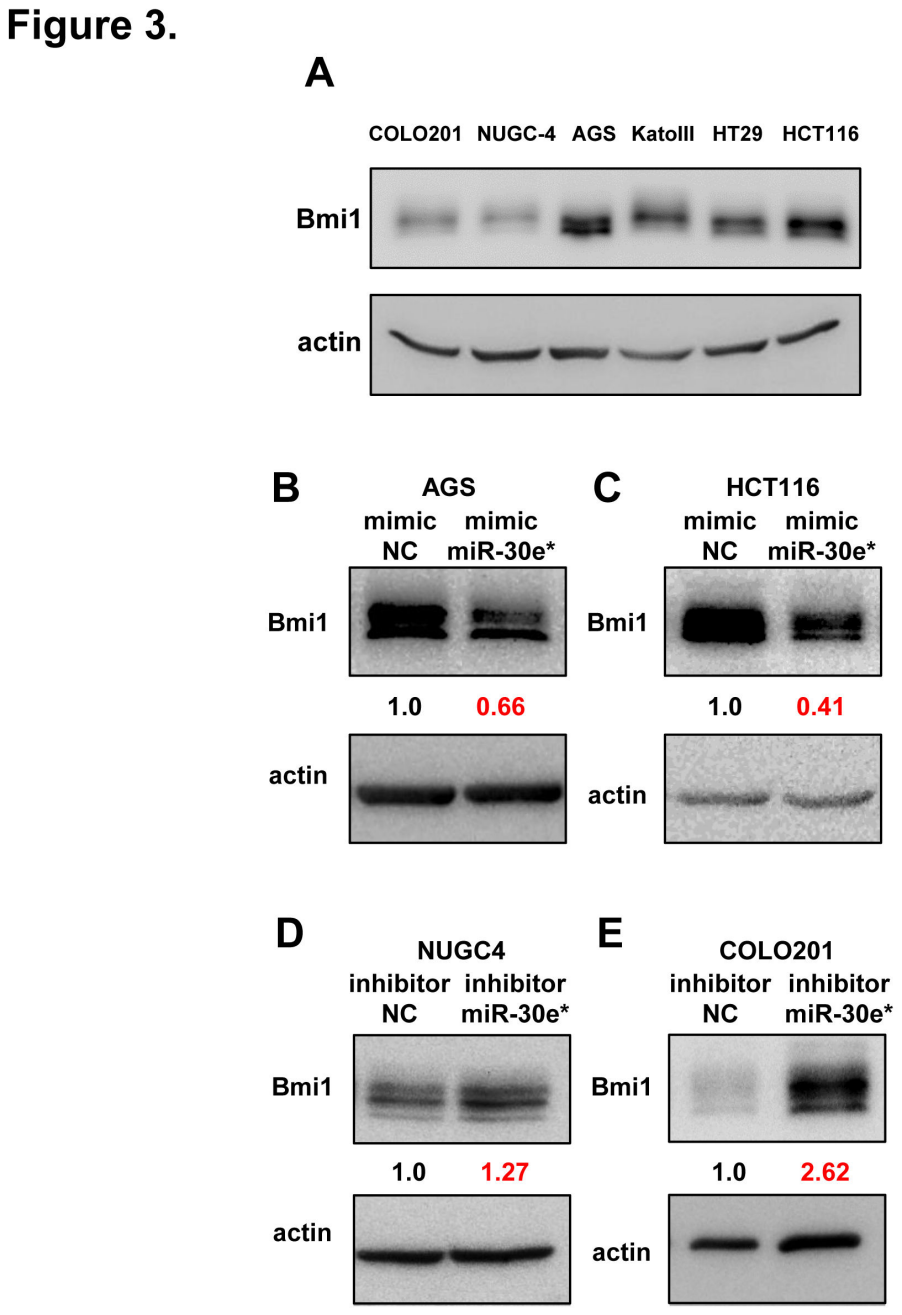
miR-30e* suppresses Bmi1 expression in gastrointestinal cells. (**A**) Western blot analysis of Bmi1 expression in 6 gastrointestinal cancer cell lines. (**B**) Western blot analysis of Bmi1 expression in high Bmi1-expressing AGS cell lines transfected with negative control (NC) and miR-30e* mimics. (**C**) Western blot analysis of Bmi1 expression in high Bmi1-expressing HCT116 cell lines transfected with NC and miR-30e* mimics. (**D**) Western blot analysis of Bmi1 expression in low Bmi1-expressing NUGC4 cell lines transfected with NC and miR-30e* inhibitors. (**E**) Western blot analysis of Bmi1 expression in low Bmi1-expressing COLO201 cell lines transfected with NC and miR-30e* inhibitors.

**Figure 4:**
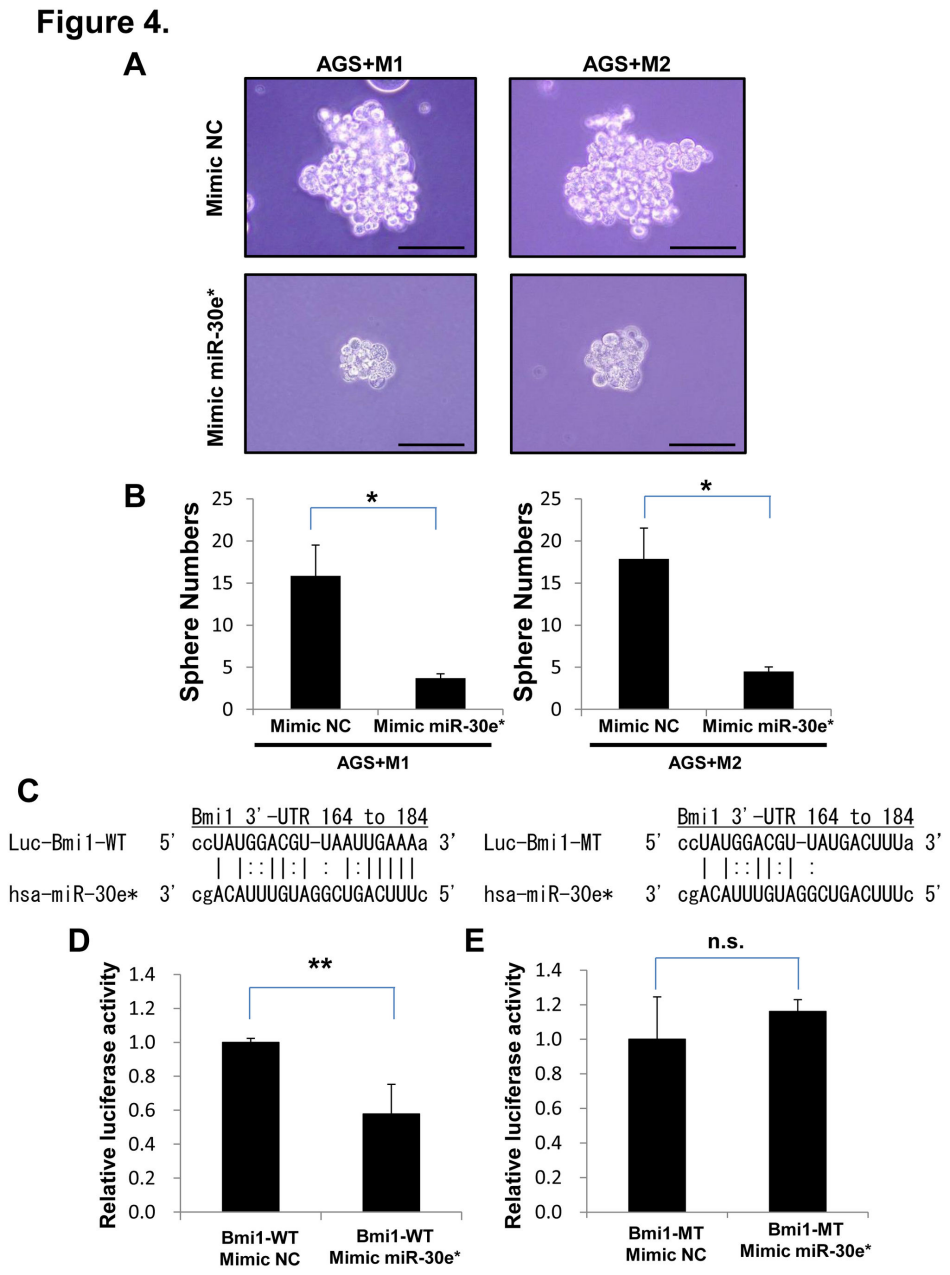
miR-30e* downregulates Bmi1 expression by directly targeting its 3′ UTR. (**A**) 3D sphere culture grown in serum-free non-adherent culture with AGS cells co-cultured with macrophages and transfected with mimic miR-30e*, compared with 3D sphere culture with AGS cells co-cultured with macrophages and transfected with mimic NC as a control group. Scale bars, 100um. (**B**) Significantly low sphere numbers were detected in mimic miR-30e* transfected groups compared with the control group (*P < 0.05, *P < 0.05, respectively). (**C**)The putative miR-30e* target site or a mutated sequence of the 3′ UTR of Bmi1 was cloned immediately downstream of the luciferase gene. (**D**) Luciferase activity of AGS cells co-transfected with plasmids containing the wild-type miR-30e* target sequence in the 3′ UTR of Bmi1 or control plasmids along with the mRNA mimic NC and mimic miR-30e*. (**E**) Luciferase activity of AGS cells co-transfected with plasmids containing the wild-type or mutant miR-30e* target sequence in the 3′ UTR of Bmi1 along with the mRNA mimic NC and mimic miR-30e*.

To reveal the functional relevance of miR-30e* expression, we examined the Bmi1 expression in the 6 gastrointestinal cancer cell lines by Western blotting (Figure 3A), and analyzed the relationship between miR-30e* and Bmi1 expression in high Bmi1 expressing cancer cell lines (AGS and HCT116) transfected with miR-30e* mimics, and low Bmi1 expressing cancer cell lines (NUGC4 and COLO201) transfected with miR-30e* inhibitors. Western blot analysis revealed significantly reduced Bmi1 protein levels in AGS and HCT116 cells transfected with miR-30e* mimics compared with controls (Figure 3B, C), and increased levels in NUGC4 and COLO201 cells transfected with miR-30e* inhibitors compared with controls (Figure 3D, E). In addition, to investigate the phenotype of cancer cells transfected with miR-30e* mimics, we performed a 3D sphere culture grown in serum-free non-adherent culture in AGS cells transfected with miR-30e* mimics (Figure 4A). The sphere formation ability of AGS cells transfected with miR-30e* mimics was inhibited (Figure 4B), so we confirmed that the downregulation of miR-30e* caused an enhanced sphere formation.

Analysis of the Bmi1 3′ UTR using the online database miRanda revealed a predicted target sequence for miR-30e*. We next investigated if miR-30e* directly targets the 3′ UTR of Bmi1 using constructs containing the putative miR-30e* target site or a mutated sequence of the 3′ UTR of Bmi1 cloned immediately downstream of a luciferase gene. The LUC-Bmi1 construct containing the predicted miR-30e* target sequence in the Bmi1 3′ UTR is shown in Figure 4C, with seed sequences indicated by lines. Transfection of AGS cells with the miR-30e* mimic significantly suppressed luciferase activity from the reporter vector containing the wild-type Bmi1 3′ UTR (LUC-Bmi1-WT) compared with the control vector (Figure 4D). We also constructed reporter vectors containing the mutant Bmi1 3′ UTR (LUC-Bmi1-MT). Transfection with the miR-30e* mimic did not suppress luciferase activity from the reporter vector containing the mutated 3′ UTR of Bmi1 compared with the wild-type 3′ UTR vector (Figure 4E). These results indicate that miR-30e* regulates Bmi1 expression by directly targeting its 3′ UTR.

### Bmi1 expression is inversely correlated with miR-30e* expression in patients with gastric cancer

**Figure 5:**
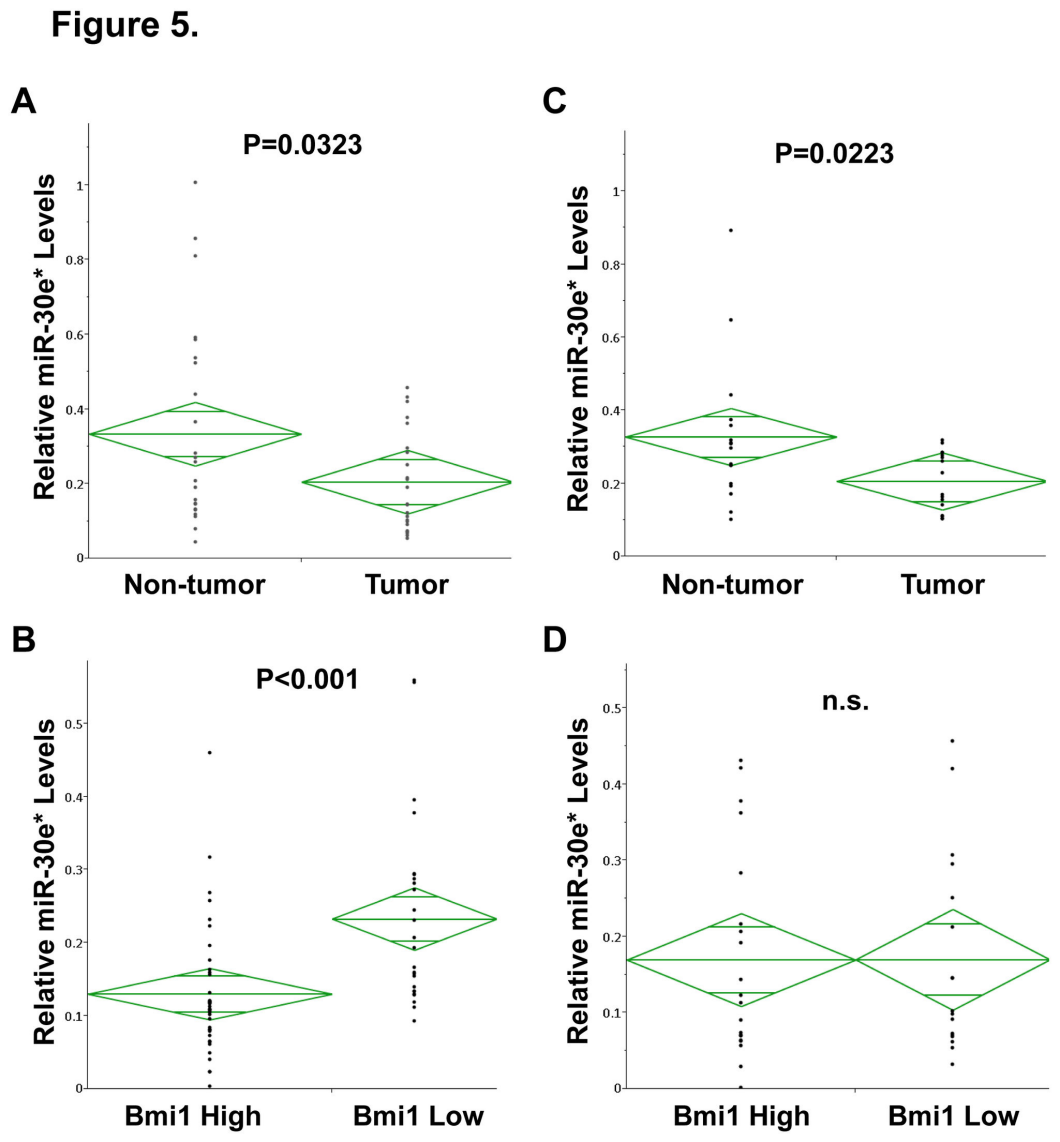
miR-30e* expression in human gastrointestinal cancer tissues. (**A**) The levels of miR-30e* expression in 16 gastric cancer tissues and their matched adjacent normal gastric epithelia as assessed by qRT-PCR. (**B**) The levels of miR-30e* expression in 29 of high and 24 of low Bmi1-expressing gastric cancer tissues as assessed by qRT-PCR. (**C**) The levels of miR-30e* expression in 37 colon cancer tissues and their matched adjacent normal colon epithelia as assessed by qRT-PCR. (**D**) The levels of miR-30e* expression in 20 of high and 17 of low Bmi1-expressing colon cancer tissues as assessed by qRT-PCR.

We next analyzed the levels of miR-30e* expression in cancer tissues and their matched adjacent normal epithelia using qRT-PCR. Expression of miR-30e* was significantly lower in cancer tissues compared with their matched adjacent normal epithelia in both gastric cancer (Figure 5A) and colon cancer (Figure 5C). Furthermore, we compared miR-30e* expression levels between high and low Bmi1 expressing cancer tissues. High Bmi1 expression levels were detected in 45% (24/53) of gastric cancer samples and 54% (20/37) of colon cancer samples. Bmi1 expression was inversely correlated with miR-30e* expression in gastric cancer (Figure 5B). However, Bmi1 expression was not associated with miR-30e* expression in colon cancer (Figure 5D). These data showed that Bmi1 expression was strongly correlated with miR-30e* expression in patients with gastric cancer but not in patients with colon cancer.

### M1- and M2-polarized macrophages purified from human monocytes induced downregulation of miR-30e* and upregulation of Bmi1

**Figure 6:**
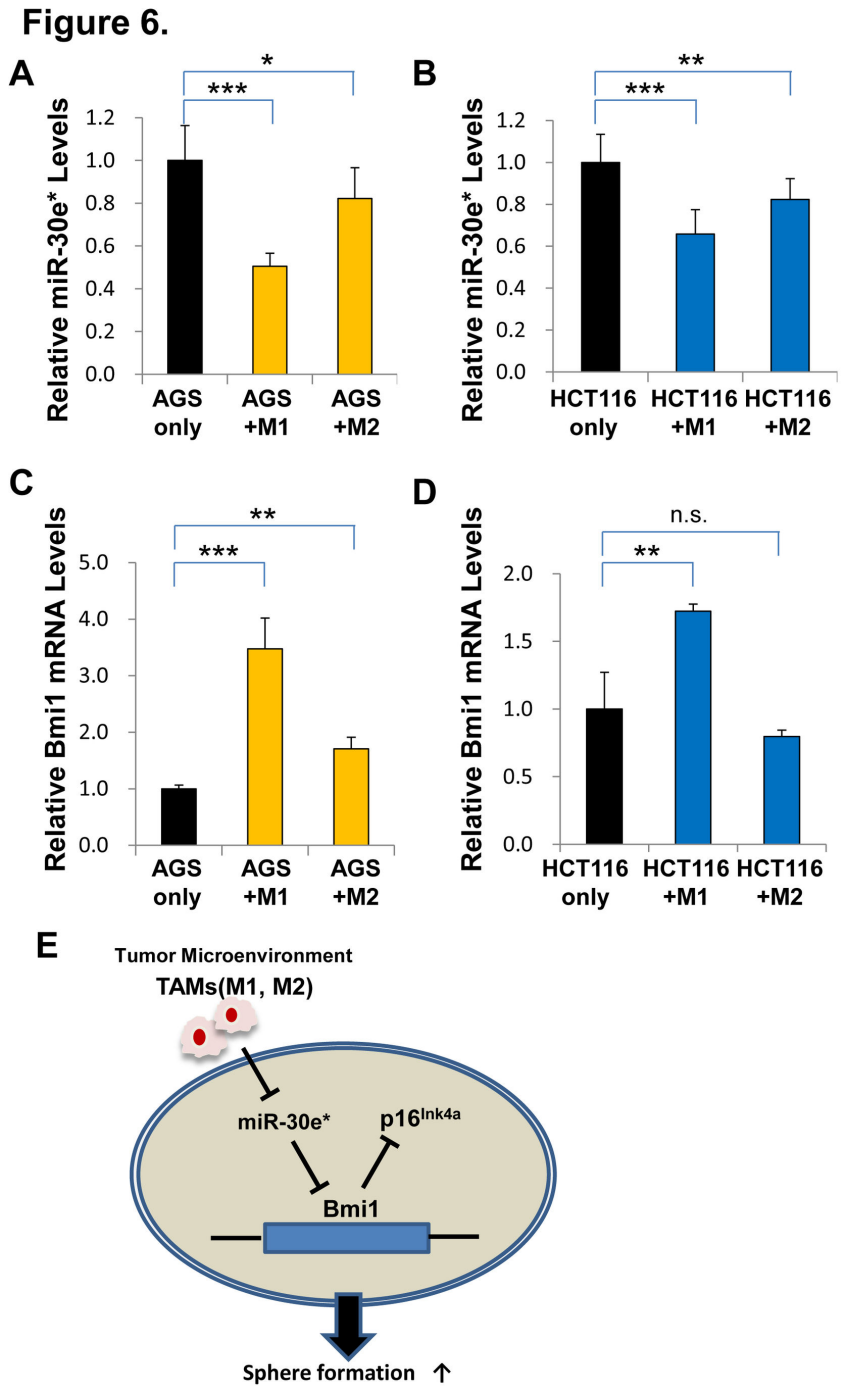
Expression of miR-30e* and Bmi1 co-cultured with M1- and M2-polarized macrophages purified from human monocytes. (**A**) qRT-PCR analysis of miR-30e* expression in AGS cells co-cultured with M1- and M2-polarized macrophages. Significantly lower miR-30e* expression was detected in co-cultured groups compared with the control group (***P < 0.001, *P < 0.05, respectively). (**B**) qRT-PCR analysis of miR-30e* expression in HCT116 cells co-cultured with M1- and M2-polarized macrophages. Significantly lower miR-30e* expression was detected in co-cultured groups compared with the control group (***P < 0.001, **P < 0.01, respectively). (**C**) qRT-PCR analysis of Bmi1 expression in AGS cells co-cultured with M1- and M2-polarized macrophages. Significantly higher Bmi1 expression was detected in co-cultured groups compared with the control group (***P < 0.001, **P < 0.01, respectively). (**D**) qRT-PCR analysis of miR-30e* expression in HCT116 cells co-cultured with M1- and M2-polarized macrophages. Significantly higher Bmi1 expression was detected in M1 macrophage co-cultured groups compared with the control group (**P < 0.01). (**E**) Schematic representation of miR-30e*-Bmi1 signaling mediated by TAMs.

Our previous results showed that Bmi1 expression was significantly increased in AGS and HCT116 cells co-cultured with both M1- and M2-polarized THP-1 macrophages. We next co-cultured AGS and HCT116 cells with M1- and M2-polarized macrophages purified from human monocytes. Bmi1 expression was significantly increased in AGS cells co-cultured with both M1- and M2-polarized macrophages purified from human monocytes, and miR-30e* expression was significantly decreased in AGS cells co-cultured with both macrophages (Figure 6A, C). In contrast, Bmi1 expression was significantly increased in HCT116 cells co-cultured with M1-polarized macrophages, but not in HCT116 cells co-cultured with M2-polarized macrophages. Expression of miR-30e* was significantly decreased in HCT116 cells co-cultured with both macrophages (Figure 6B, D). This result demonstrated that M1- and M2-polarized macrophages purified from human monocytes induced downregulation of miR-30e* in gastrointestinal cell lines, and upregulation of Bmi1 in gastric cancer cell line, but not in colon cancer cell line.

## Discussion

In this study, we showed that TAMs upregulated Bmi1 expression, leading to increased sphere formation ability. Bmi1 expression was suppressed by miR-30e* through miR-30e* direct binding to Bmi1 3′ UTR, and Bmi1 expression was inversely correlated with miR-30e* expression in cancer tissues. Gastrointestinal cells co-cultured with macrophages purified from human monocytes showed increased Bmi1 expression. Together these data demonstrate that TAMs regulate miR-30e* targeting of Bmi1 in gastrointestinal cancer cells.

Previous reports have shown that TAMs contribute to tumor progression through secretion EGF and upregulation of the EGFR/Stat3/Sox-2 signaling pathway [24]. We demonstrated that TAMs upregulated Bmi1 expression and enhanced sphere formation. Our findings suggest that Bmi1 upregulation enhanced sphere formation, possibly through suppression of the INK4a-ARF locus. 

In this study, we demonstrated that Bmi1 expression and sphere formation ability were significantly increased in AGS and HCT116 cells co-cultured with both M1- and M2-polarized THP-1 macrophages. Previous studies showed that M2-polarized macrophages promote the growth and vascularization of tumors, while M1-polarized macrophages have tumoricidal activity. So, in many human cancers, it has been proposed that TAMs were predominantly polarized to M2 macrophage phenotype [13-17]. However, other studies showed that the degree of M2 macrophage infiltration was very correlative with a better prognosis in gastrointestinal cancer [25,26]. Thus, it remains controversial which macrophages (M1 or M2) promote tumor progression in gastrointestinal cancer. Furthermore, more recent studies showed that macrophages were plastic, and their epigenetic changes reprogramed TAMs from an M2 to an M1-like phenotype in tumors [17,18]. So we speculated that TAMs were not predominantly polarized to M2 macrophage phenotype in gastrointestinal cancer.

Bmi1 is regulated by Twist1 which is one of the epithelial mesenchymal transition inducers in head and neck cancer cells [27]. In breast cancer cells, Bmi1 activates the WNT pathway by repressing the expression of DKK family members, leading to increased c-Myc, which upregulates Bmi1 via a c-Myc binding site [28]. In colon cancer cells, Bmi1 is directly suppressed by KLF4 [29]. However, the mechanisms underlying Bmi1 regulation have not been completely clear.

Several recent studies have uncovered evidence for microRNA-mediated regulation of Bmi1. miR-128a increases intracellular ROS levels by targeting Bmi1, resulting in inhibition of cancer cell growth in medulloblastoma cells. Both miR-15b and miR-200b regulate chemotherapy-induced EMT by downregulating Bmi1 in tongue squamous cell carcinomas, and miR-218 inhibits cell proliferation and cycle progression and promotes apoptosis by downregulating Bmi1 in colorectal cancer cells [30-32]. In this study, we selected microRNAs that were downregulated in cancer cell lines co-cultured with TAMs compared with controls, and identified miR-30e* as binding Bmi1 3ʹ UTR, using *in silico* prediction methods. miR-30e* was recently shown to be downregulated in various cancers. miR-30e* directly binds PIK3C2A 3ʹ UTR in colorectal cancer and IκBα 3ʹ UTR in glioma [33,34]. However, no evidence for Bmi1 regulation by miR-30e* had been previously reported. Our results showed that suppression of miR-30e* increased Bmi1 expression, and that overexpression of miR-30e* decreased Bmi1 expression. In addition, our luciferase assay demonstrated that miR-30e* directly targeted the 3′ UTR of Bmi1.

We also investigated the relationship between miR-30e* and Bmi1 expression in clinical samples. miR-30e* expression was decreased in tumor regions compared with non-tumor regions in gastrointestinal cancer, and miR-30e* expression was inversely correlated with Bmi1 expression in gastric cancer, but not in colon cancer. It may be possible that another pathway, such as the Wnt pathway or KLF4, plays a more important role in the upregulation of Bmi1 expression than miR-30e* in colon cancer.

In addition, we performed a co-cultured assay with macrophages purified from human monocytes. The changes of miR-30e* and Bmi1 expression were more dominant in cells co-cultured with M1 macrophages. In macrophages purified from human monocytes, M1 macrophages secrete IL-6, TNF-α, and IL-1β, while M2 macrophages secrete TGF-β and IL-10 [13]. On the other hand, in THP-1 derived M1 macrophages, IL-6, TNF-α, and IL-1β are released at higher levels compared with M2, while M2 macrophages secrete more TGF-β than M1 macrophages [23]. The cytokine profile produced by both THP-1 macrophages and macrophages purified from human monocytes are very similar, but production levels of the cytokines may differ as THP-1 macrophages are from a leukemia cell line, while macrophages purified from human monocytes are primary culture cells. These results showed that distinct cytokines profiles in M1 and M2 macrophages, and the discrepancy may influence miR-30e* and Bmi1 expression. Next, we assessed the different relations between Bmi1 and miR-30e* expression in gastric and colon cancer cell lines which were co-cultured with macrophages purified from human monocytes. Previous studies showed that Wnt signaling is implicated in self-renewal activity of colon cancer cells, while Bmi1 is regulated by Wnt signaling in breast cancer cells [28,35]. It may be possible that another pathway such as the Wnt pathway has more impact on the Bmi1 regulation than miR-30e* pathway in colon cancer cell lines, as well as in colon clinical samples. Furthermore, we speculated that the cytokine which suppresses miR-30e* expression could be derived from M1 macrophages, and thus performed Bmi1 expression in cancer cells treated with these cytokines produced by M1 macrophages. But the qRT-PCR analysis showed that Bmi1 expression was not significantly increased in AGS cells treated with these cytokines. We could not identify the cytokine that suppress miR-30e*. Therefore, more analysis is required to determine the underlying mechanism.

 In conclusion, we identified that TAMs could promote gastrointestinal cancer, especially gastric cancer, progression by downregulating miR-30e* and upregulating Bmi1. These findings suggest that the suppression of TAMs induced Bmi1 expression could be a possible treatment strategy for gastrointestinal cancer.
